# Late Breaking News

**DOI:** 10.1111/ene.70263

**Published:** 2025-06-21

## Monday, June 23 2025

## LBN_01

### Achieving higher standards in real‐world migraine care with anti‐CGRP monoclonal antibodies

#### E. Caronna ^1^; R. Mas‐de‐les‐Valls^2^; C. Sundal^3^; P. Irimia^4^; A. Lozano Ros^5^; A. Gago‐Veiga^6^; F. Velasco Juanes^7^; R. Ruscheweyh^8^; R. Gil‐Gouveia^9^; M. Huerta‐Villanueva^10^; J. Rodriguez‐Vico^11^; J. Viguera Romero^12^; V. Obach^13^; S. Santos‐Lasaosa^14^; M. Ghadiri‐Sani^15^; C. Tassorelli^16^; J. Díaz de Terán^17^; S. Díaz Insa^18^; C. González Oria^19^; S. Sacco^20^; E. Cuadrado‐Godia^21^; D. García‐Azorín^22^; J. Pascual^23^; P. Barbanti^24^; P. Pozo‐Rosich^1^

##### ^*1*^ *Headache Clinic, Neurology Department, Vall d’Hebron Hospital, Barcelona, Spain;* ^*2*^ *Headache and Neurological Pain Research Group, VHIR, Department of Medicine, Universitat Autònoma de Barcelona, Barcelona, Spain;* ^*3*^ *NeuroClinicNorway, Department of Neurology, Norway;* ^*4*^ *Department of Neurology, Clinica Universidad de Navarra, Pamplona, Spain;* ^*5*^ *Headache Unit, Hospital General Universitario Gregorio Marañón, Madrid, Spain;* ^*6*^ *Headache Unit, Neurology Department, Hospital Universitario La Princesa, Madrid, Spain;* ^*7*^ *Neurology Department, Hospital Universitario Cruces, Biocruces Bizkaia Health Research Institute, Bilbao, Spain;* ^*8*^ *Department of Neurology, LMU University Hospital, LMU Munich, Munich, Germany;* ^*9*^ *Hospital da Luz Headache Center, Neurology Department, Hospital da Luz – Lisboa, Lisbon, Portugal;* ^*10*^ *Headache Unit, Neurology Department, Hospital Universitari de Bellvitge‐IDIBELL, Spain;* ^*11*^ *Headache Unit, Hospital Universitario Fundación Jiménez Díaz, Madrid, Spain;* ^*12*^ *Headache Unit, Hospital Virgen Macarena, Seville, Spain;* ^*13*^ *Hospital Clinic, Barcelona, Spain;* ^*14*^ *Neurology Department, Hospital Clínico Universitario Lozano Blesa, Zaragoza, Spain;* ^*15*^ *The Walton Centre NHS Foundation Trust, Liverpool, UK;* ^*16*^ *Headache Science & Neurorehabilitation Center, IRCCS Mondino Foundation, Pavia, Italy;* ^*17*^ *Headache Unit, Neurology Department, La Paz University Hospital, Madrid, Spain;* ^*18*^ *Headache Unit, Neurology, Hospital Universitari i Politècnic La Fe, Valencia, Spain;* ^*19*^ *Unidad de Cefaleas, Hospital Universitario Virgen del Rocío, Sevilla, Spain;* ^*20*^ *Department of Biotechnological and Applied Clinical Sciences, University of L’Aquila, L’Aquila, Italy;* ^*21*^ *Neuroscience Research Program, Hospital del Mar Research Institute, Pompeu Fabra University, Spain;* ^*22*^ *Headache Unit, Department of Neurology, Hospital Clínico Universitario de Valladolid, Spain;* ^*23*^ *University Hospital Marqués de Valdecilla, Universidad de Cantabria, Santander, Spain;* ^*24*^ *Headache and Pain Unit, IRCCS San Raffaele, Roma, Italy; Italian Migraine Registry (IGRAINE) study group*

**Background and Aims:** The International Headache Society has proposed new treatment goals for migraine prevention in real world, as a way to set higher standards of care. This study provides the first assessment of the proportion of individuals achieving them after 6 months of migraine‐specific treatment with anti‐CGRP monoclonal antibodies (MAbs).

**Methods:** European multicenter, prospective, real‐world study, including adults with migraine treated with anti‐CGRP MAbs (EUREkA cohort). We assessed the proportions of individuals in each treatment goal category – migraine freedom (0 monthly migraine days – MMD); optimal control (≤4 MMD), modest control (4–6 MMD); insufficient control (>6 MMD) – after 6 months of treatment. We also assessed the proportions of individuals with ≥50% reduction in monthly headache days – MHD in the insufficient control group.

**Results:** 4963 had 6 months data: 82.3% (4086/4963) were female and median age was 48.0 [40.0–55.0] years. At baseline, the median MHD, MMD were 20.0 [13.3–28.0] days/months and 15.0 [10.0–20.0] days/months, respectively. All the cohort at baseline was classified as having insufficient control. At month 6, 6.9% (342/4963) had migraine freedom, 32.0% (1589/4,963) optimal control, 15.5% (771/4963) modest control and 45.6% (2261/4963) insufficient control. In the insufficient control group, 27.1% (613/2261) of individuals were ≥50% responders.

**Conclusion:** High standards of care, defined as optimal disease control or even migraine freedom, are achieved in real‐world settings with anti‐CGRP MAbs in approximately 40% of individuals with a high migraine burden. These findings highlight the need to expand global access to these treatments.

**Disclosure:** EC has received honoraria from Novartis, Chiesi, Lundbeck, MedScape; his salary has been partially funded by Río Hortega grant Acción Estratégica en Salud 2017–2020, Instituto de Salud Carlos III (CM20/00217). He is a junior editor for Cephalalgia. PPR has received, in the last three years, honoraria as a consultant and speaker for: AbbVie, Dr Reddy's, Eli Lilly, Lundbeck, Medscape, Novartis, Organon Pfizer and Teva. Her research group has received research grants from AbbVie, Novartis and Teva; as well as, Instituto Salud Carlos III, EraNet Neuron, European Regional Development Fund (001‐P‐001682) under the framework of the FEDER Operative Programme for Catalunya 2014‐2020 – RIS3CAT; has received funding for clinical trials from AbbVie, Amgen, Biohaven, Eli Lilly, Novartis, Pfizer, Teva. She is the Honorary Secretary of the International Headache Society. She is in the editorial board of Revista de Neurologia. She is an associate editor for Cephalalgia, Headache, Neurologia, The Journal of Headache and Pain and Frontiers of Neurology. She is a member of the Clinical Trials Guidelines Committee of the International Headache Society. She has edited the Guidelines for the Diagnosis and Treatment of Headache of the Spanish Neurological Society. She is the founder of www.midolordecabeza.org. PPR does not own stocks from any pharmaceutical company.

## LBN_02

### Shared neural signatures of photophobia in migraine and post‐traumatic headache: A task‐based fMRI study

#### R. Häckert Christensen; H. M. Al‐Khazali; A. Melchior; M. Ashina; H. Ashina

##### Department of Neurology, Danish Headache Center, Copenhagen University Hospital – Rigshospitalet, Copenhagen, Denmark

**Background and Aims:** Persistent post‐traumatic headache (PPTH) and migraine frequently present with photic hypersensitivity that exacerbates headache symptoms. We sought to determine whether PPTH is associated with altered brain responses to visual stimuli and to explore shared neural mechanisms of photophobia with migraine.

**Methods:** This cross‐sectional functional magnetic resonance imaging (fMRI) study included 80 adults with PPTH, 261 with migraine, and 143 healthy controls (HCs). All participants underwent visual stimulation using a flickering checkerboard during a 3T fMRI session. Blood oxygen level‐dependent (BOLD) responses were examined using whole‐brain and region‐of‐interest (ROI) analyses. All analyses were adjusted for age and sex.

**Results:** Whole‐brain analysis revealed no significant BOLD differences across the full PPTH, migraine, and HC groups. However, participants with PPTH who experienced photophobia (*n* = 41) showed greater activation in the anterior and mid‐cingulate cortex compared with HCs (PFWE = 0.010). No differences were observed between photophobic participants with PPTH and those with migraine who reported an attack during the fMRI session. ROI analyses identified greater activation in the anterior cingulate, mid‐cingulate, and insular cortices in both photophobic participants with PPTH and ictal participants with migraine, relative to HCs (all *p* < 0.05). No significant differences were found between photophobic participants with PPTH and ictal participants with migraine.

**Conclusion:** Photophobia in persistent PPTH is associated with greater activation in cortical regions implicated in pain processing. These patterns parallel those observed during migraine attacks, indicating shared neural mechanisms between the two headache disorders.

**Disclosure:** This work received support by research grants from the Lundbeck Foundation (R403‐2022‐1352 and R310‐2018‐3711).

## LBN_03

### Increased serum neurofilament light chain levels in Parkinson's disease patients carrying the p.A53T SNCA mutation

#### N. Papagiannakis ^1^; C. Koros^1^; A. Simitsi^1^; D. Lafontant^2^; K. Marek^3^; A. Siderowf^4^; T. Simuni^5^; L. Stefanis^1^

##### ^*1*^ *1st Department of Neurology, Eginiteio Hospital, National and Kapodistrian University of Athens, Athens, Greece;* ^*2*^ *Department of Biostatistics, College of Public Health, University of Iowa, Iowa City, USA;* ^*3*^ *Institute for Neurodegenerative Disorders, New Haven, USA;* ^*4*^ *Department of Neurology, University of Pennsylvania Perelman School of Medicine, Philadelphia, USA;* ^*5*^ *Parkinson's Disease and Movement Disorders Center, Northwestern University Feinberg School of Medicine, Chicago, USA*

**Background and Aims:** Neurofilament light chain (NfLs) is an intermediate filament neuronal‐specific cytoskeletal axonal protein, whose blood and CSF levels are increased in various neurodegenerative diseases, reflecting neuro‐axonal degeneration; in uncomplicated Parkinson's Disease (PD) however, such levels are within normal limits. A53T‐PD, due to the p.A53T mutation in the SNCA gene, is a severe and rapidly progressive genetic synucleinopathy. We report here serum NfL measurements in this rare condition.

**Methods:** Serum NfL and demographic data were acquired from Parkinson's Progression Markers Initiative (PPMI) database. A propensity score matching based technique was used to match PD patients without genetic causes (iPD) and healthy controls, with A53T‐PD cases, in a 3:1 ratio (i.e. 3 iPD and 3 controls for every A53T‐PD case). The logistic regression model for the matching score used the age and age of onset (in the case of iPD) as independent variables, and it was fed in a nearest neighbor matching algorithm.

**Results:** Serum NfL data were available for 18 A53T‐PD cases. Consequently, 54 iPD and 54 controls were selected by the matching algorithm. NfL levels were increased in A53T‐PD (13.8 pg/mL) in comparison with iPD (7.56 pg/mL) and healthy controls (8.01 pg/mL) (overall *p* = 0.010).

**Conclusion:** The increase in serum NfL points to a more aggressive neurodegenerative process in A53T‐PD compared to iPD.

**Disclosure:** This study was funded by the Michael J Fox Foundation through the Write Now initiative.

## LBN_04

### Efficacy and safety of efgartigimod as an add‐on therapy in patients with NMOSD and MOGAD

#### W. Yin^1^; S. Wang^2^; W. Lu^1^; T. Duan ^1^

##### ^*1*^ *Department of Neurology, the Second Xiangya Hospital, Central South University, Changsha, Hunan, China;* ^*2*^ *Faculty of Biology, Medicine and Health, University of Manchester, Michael Smith Building, Manchester, UK*

**Background and Aims:** Neuromyelitis optica spectrum disorders (NMOSD) and myelin oligodendrocyte glycoprotein associated disease (MOGAD) are autoimmune antibody mediated diseases. Efgartigimod is a neonatal Fc receptor targeting therapeutic, causing antibody titers reduction. The efficacy and safety of efgartigimod added with intravenous methylprednisolone (IVMP) in NMOSD and MOGAD patients was assessed in this study.

**Methods:** Patients diagnosed with NMOSD or MOGAD were enrolled. Efgartigimod was administered intravenously 10 mg/kg × 4 doses or 20 mg/kg × 2 doses. Expanded disability status scores (EDSS), serum immunoglobulin G (IgG) levels and pathogenic antibody titers were evaluated before and after therapy.

**Results:** In total, 27 adult patients were enrolled, 13 patients were treated with IVMP plus efgartigimod, comparable 14 controls treated with IVMP alone. Efgartigimod group achieved better outcome with EDSS reduction by 1.3 ± 0.6, compared with control group (0.5 ± 0.5). Meanwhile, serum IgG levels in efgartigimod treated group decreased by 69.8% after therapy, and nine patients (69.2%) showed antibody titers reduction. Moreover, the EDSS and antibody titers decreased more rapidly and largely in intensive therapy cohort (20 mg/kg ×2).

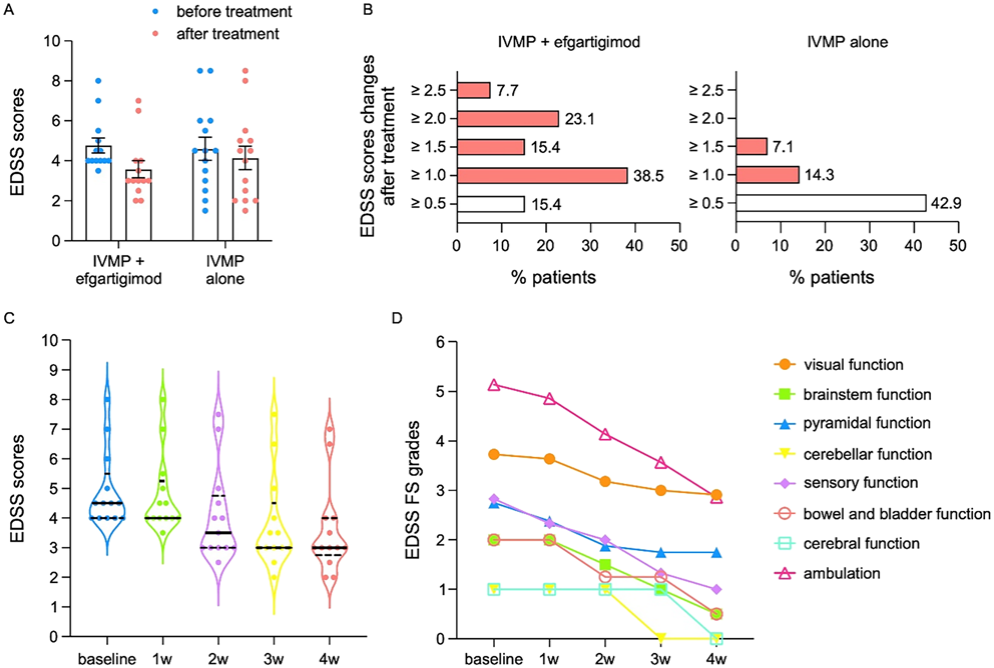

**FIGURE 1** The changes of EDSS scores in IVMP plus efgartigimod group and in IVMP alone group before and after treatment.

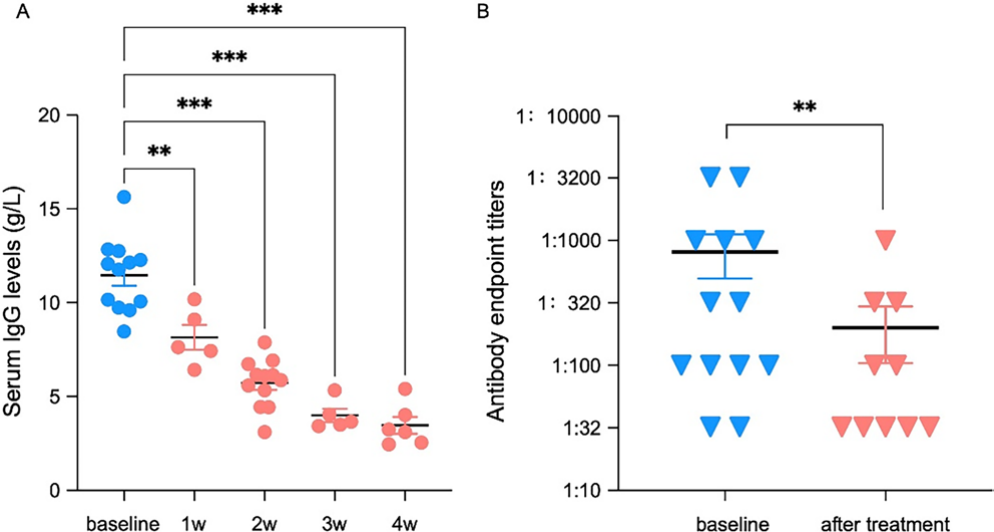

**FIGURE 2** Levels of serum IgG and serum pathogenic antibody titers at baseline and after treatment of efgartigimod in NMOSD and MOGAD patients.

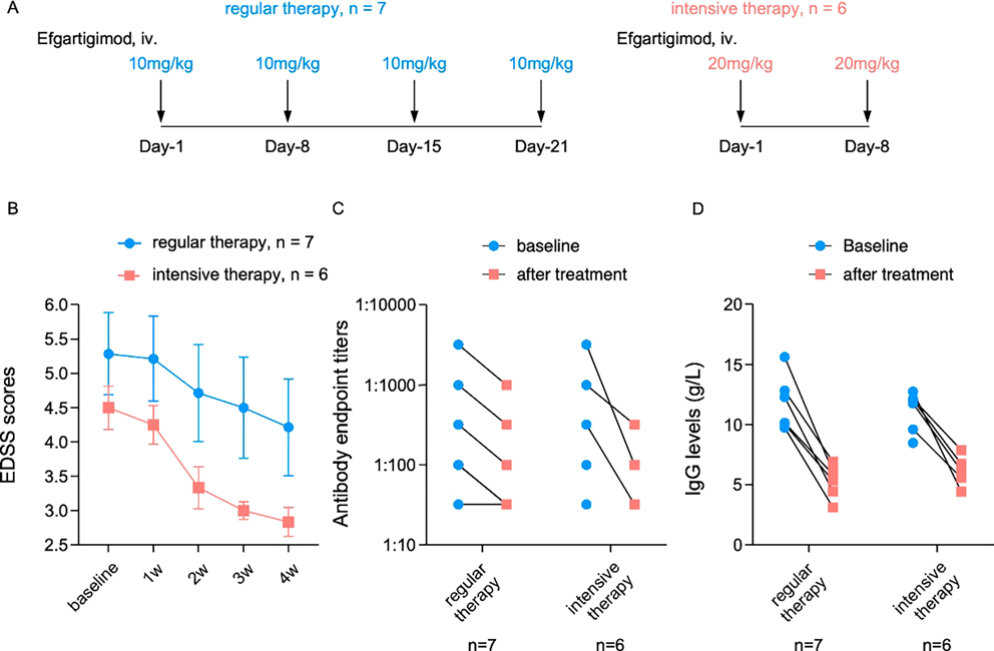

**FIGURE 3** The efficacy of efgartigimod in regular and intensive therapy cohort.

**Conclusion:** Efgartigimod add‐on therapy was beneficial and tolerable in patients with NMOSD and MOGAD in acute phase.

**Disclosure:** Nothing to disclose.

## LBN_05

### Detection of misfolded TDP‐43 in CSF of genetic FTD and FTD/ALS patients at both presymptomatic and symptomatic stages

#### P. Caroppo ^1^; I. Dellarole^2^; V. Aprea^1^; M. Catania^1^; C. Battipaglia^1^; A. Romeo^1^; C. Villa^1^; E. Dalla Bella^3^; N. Riva^3^; G. Rossi^1^; G. Di Fede^1^; G. Legname^4^; J. De Houwer^5^; A. Alberici^6^; B. Borroni^7^; J. van Swieten^5^; F. Moda^8^

##### ^*1*^ *Neurology 8 – Dementias and degenerative diseases of CNS Unit, Fondazione IRCCS Istituto Neurologico Carlo Besta, Milan, Italy;* ^*2*^ *SSD Laboratory Medicine‐Laboratory of Clinical Pathology, Fondazione IRCCS Istituto Neurologico Carlo Besta, Milan, Italy;* ^*3*^ *Neurology 3 – Neuroalgology Unit, Fondazione IRCCS Istituto Neurologico Carlo Besta, Milan, Italy;* ^*4*^ *Laboratory of Prion Biology, Department of Neuroscience, Scuola Internazionale Superiore Di Studi Avanzati (SISSA), Trieste, Italy;* ^*5*^ *Department of Neurology and Alzheimer Centre, Erasmus MC University Medical Centre (Erasmus MC), Rotterdam, The Netherlands;* ^*6*^ *Department of Continuity of Care and Frailty, ASST Spedali Civili Brescia Hospital, Brescia, Italy;* ^*7*^ *Department of Clinical and Experimental Sciences, University of Brescia, Brescia, Italy; Molecular Markers Laboratory, IRCCS Istituto Centro San Giovanni di Dio Fatebenefratelli, Brescia, Italy;* ^*8*^ *SSD Laboratory Medicine‐Laboratory of Clinical Pathology, Fondazione IRCCS Istituto Neurologico Carlo Besta, Milan, Italy; Department of Medical Biotechnology and Translational Medicine, University of Milan, Milan, Italy*

**Background and Aims:** Seed amplification assays (SAAs) have shown promising results in detecting misfolded TDP‐43 in cerebrospinal fluid (CSF) of patients with genetic FTD with TDP‐43 pathology. However, there are no data available on SAA analysis of CSF from subjects in the presymptomatic phase of the disease.

**Methods:** We tested TDP‐43 seeding activity in CSF of 32 patients carrying pathogenic GRN mutations, C9orf72 expansion and MAPT mutations, 14 presymptomatic carriers and 12 controls (subjects without neurodegenerative disorders). Truncated recombinant TDP‐43 protein has been used as a SAA reaction substrate. We also used Single Molecule Array technology for measuring the neurofilament light chain (NfL) levels in the entire cohort.

**Results:** We found a seeding activity in in 67% of TDP‐43‐linked symptomatic patients, with a specificity of 93%. A half of presymptomatic subjects tested positive, most of them GRN carriers. Interestingly, among TDP‐43_SAA positive presymptomatic individuals, two GRN carriers underwent phenoconversion. Furthermore, presymptomatic individuals who tested positive for TDP‐43_SAA had also higher levels of NfL compared to TDP‐43 negative individuals.

**Conclusion:** We confirm the presence of seeding activity for TDP‐43 in the CSF of symptomatic patients with genetic forms of TDP‐43 pathology. However, what is particularly intriguing is our demonstration that this seeding activity is also detectable in presymptomatic disease stages, mostly in GRN mutation carriers. We also suggest a possible link between positive TDP‐43_SAA and conversion to the symptomatic phase.

**Disclosure:** The work was supported by the project JPND – MINDFACE – MIcroglial early Neuroinflammatory Dysfunction in Frontotemporal Dementia and Amyotrophic Lateral Sclerosis due to C9orf72 repeat Expansions (Coordinator, Prof Van Swieten). Paola Caroppo has received funding from Italian Ministry of Health.

## LBN_06

### Early Versus Delayed Add‐on Therapy in Generalised Myasthenia Gravis: A Multicentre Real‐World Cohort Study

#### M. Oeztuerk ^1^; N. Huntemann^2^; L. Gerischer^3^; A. Meisel^3^; C. Nelke^1^; F. Stascheit^3^; C. Menskes^2^; S. Meuth^2^; S. Lehnerer^3^; S. Pfeuffer^4^; H. Krämer‐Best^4^; T. Hagenacker^5^; T. Ruck^1^

##### ^*1*^ *Ruhr University Bochum, BG University Hospital Bergmannsheil, Department of Neurology, Bochum, Germany;* ^*2*^ *Department of Neurology, Medical Faculty, Heinrich Heine University Düsseldorf, Düsseldorf, Germany;* ^*3*^ *Department of Neurology, Charité — Universitätsmedizin Berlin, Corporate Member of Freie Universität Berlin, Humboldt‐Universität zu Berlin, Berlin, Germany;* ^*4*^ *Department of Neurology, Justus Liebig University of Giessen, Giessen, Germany;* ^*5*^ *Department of Neurology and Center for Translational Neuro‐ and Behavioral Sciences (C‐TBNS), University Medicine Essen, Essen, Germany*

**Background and Aims:** Generalised myasthenia gravis (gMG) is an autoimmune disorder marked by fluctuating skeletal muscle weakness due to antibodies targeting the neuromuscular junction. Although many patients respond to standard immunosuppression, a substantial subgroup experiences persistent symptoms or medication‐related toxicity. Recently approved add‐on therapies – complement component 5 inhibitors (C5IT) and neonatal Fc receptor (FcRn) antagonists – offer new treatment avenues. However, the optimal timing of therapy escalation remains unclear.

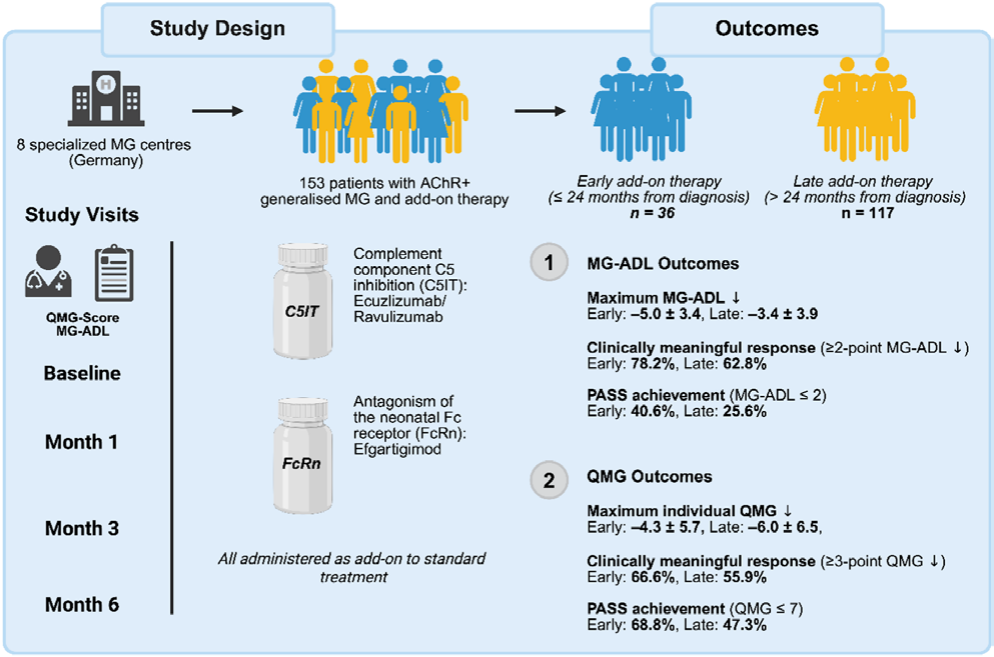

**FIGURE 1** Study overview.

**Methods:** In this multicentre, retrospective cohort study, 153 patients with acetylcholine receptor antibody‐positive gMGs were treated at eight specialised German centres with either C5IT (eculizumab or ravulizumab) or FcRn antagonism (efgartigimod). Patients were grouped based on whether treatment began within (*n* = 36) or after (*n* = 117) 24 months of diagnosis. Disease severity was assessed at baseline and at 1, 3, and 6 months using the Myasthenia Gravis Activities of Daily Living (MG‐ADL) and Quantitative Myasthenia Gravis (QMG) scores. Primary outcome was maximum MG‐ADL improvement. Secondary endpoints included clinically meaningful response (≥2‐point MG‐ADL reduction), PASS (MG‐ADL ≤2), MSE (MG‐ADL ≤1), and QMG‐based response (≥3‐point reduction).

**Results:** Early‐treated patients showed greater improvements: 78.2% vs. 62.8% achieved MG‐ADL response; 40.6% vs. 25.6% reached PASS; 21.9% vs. 18.0% met MSE; and 66.6% vs. 55.9% showed QMG response. QMG worsening occurred in 6.3% (early) vs. 16.2% (late).

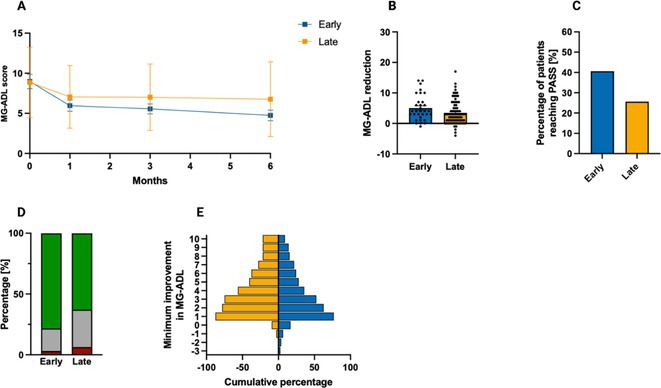

**FIGURE 2** MG‐ADL outcomes in patients undergoing early versus late treatment escalation.

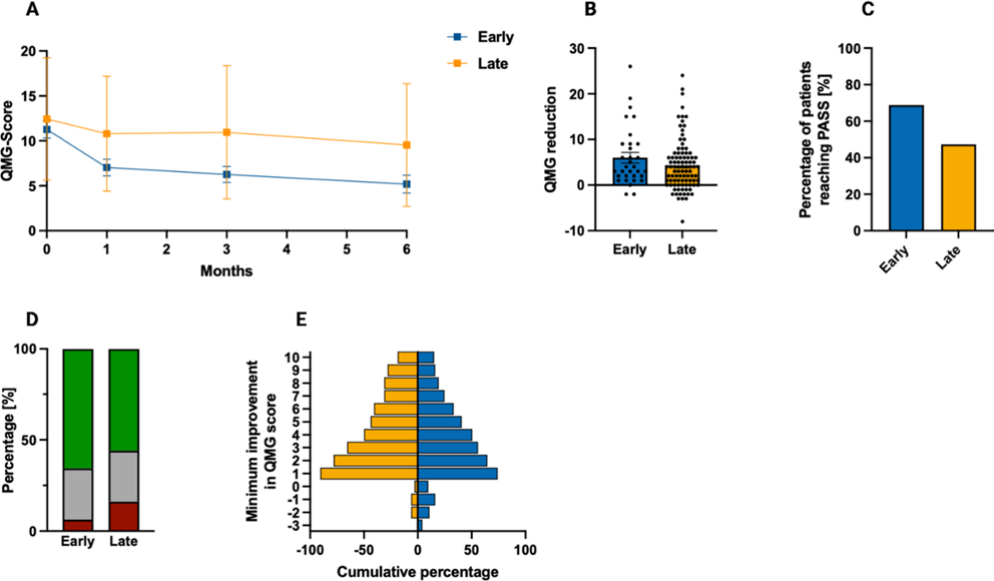

**FIGURE 3** QMG outcomes in patients undergoing early versus late treatment escalation.

**Conclusion:** Early initiation of add‐on therapy was associated with more favourable outcomes, supporting a time‐sensitive treatment approach in gMG.

**Disclosure:** Nothing to disclose.

